# FEELINGS OF BURDEN AMONG OLDER CHILDREN OF VERY OLD PARENTS IN THE US AND KOREA

**DOI:** 10.1093/geroni/igac059.484

**Published:** 2022-12-20

**Authors:** Kyungmin Kim, Yijung Kim, Joohyun Kim, Kathrin Boerner, Daniela Jopp, Gyounghae Han

**Affiliations:** Seoul National University, Seoul, Seoul-t'ukpyolsi, Republic of Korea; The University of Texas at Austin, Austin, Texas, United States; Seoul National University, Seoul, Seoul-t'ukpyolsi, Republic of Korea; University of Massachusetts Boston, Boston, Massachusetts, United States; University of Lausanne, Lausanne, Vaud, Switzerland; Seoul National University, Seoul, Seoul-t'ukpyolsi, Republic of Korea

## Abstract

Very old adults (80+) are the fastest growing population worldwide. Children of the very old adults may see their prolonged relationship with parents as a benefit (e.g., longer time together) but also as burden (e.g., prolonged responsibility in their own late life). Using a sample of older children (N = 219) from the Boston Aging Together Study and Korean Aging Together Study, we investigated the factors associated with older children’s (aged 62–76) reports of burden in their relationships with very old parents (aged 81–101), focusing on how family relations were imbedded in different cultural contexts. Overall, American older children showed lower levels of burden, compared to Korean older children. The factors associated with burden differed by country; support given to parents and relationship quality were associated with American older children’s burden, whereas support received from parents, familism, and negative relationship quality were associated with Korean older children’s burden.

